# Circulating biomarkers improve prediction of postoperative outcome after aortic valve surgery

**DOI:** 10.3389/fcvm.2026.1813821

**Published:** 2026-06-02

**Authors:** Till Joscha Demal, Jenny Bialczak, Alina Goßling, Francisco Miguel Ojeda, Oliver Daniel Bhadra, Björn Sill, Johannes Petersen, Sebastian Ludwig, David Grundmann, Lisa Voigtländer, Lara Waldschmidt, Laura Hannen, Niklas Schofer, Stefan Blankenberg, Paulus Kirchhof, Thomas Renné, Lenard Conradi, Hermann Reichenspurner, Evaldas Girdauskas, Andreas Schaefer

**Affiliations:** 1 Department of Cardiovascular Surgery, University Heart and Vascular Center Hamburg, Hamburg, Germany; 2 German Center for Cardiovascular Research, partner site Hamburg/Kiel/Lübeck, Hamburg, Germany; 3 Department of Cardiology, University Heart and Vascular Center Hamburg, Hamburg, Germany; 4 Department of Clinical Chemistry and Laboratory Medicine, University Medical Center Hamburg-Eppendorf, Hamburg, Germany

**Keywords:** aortic valve disease, biomarkers, euro SCORE II, risk stratificacion, surgical aortic valve replacement (SAVR)

## Abstract

**Background:**

Predicting adverse outcomes following aortic valve surgery remains challenging. This study aimed to investigate the association between preoperative circulating biomarkers and postoperative mortality and morbidity, with the goal of improving established risk stratification tools.

**Methods:**

Between March 2018 and May 2022, 492 patients underwent surgical aortic valve replacement or repair and were included in a registry. Preoperative blood biomarkers, including hemoglobin, creatinine, high-sensitivity troponin I (hsTrop-I), GOT, GPT, INR, CRP, NT-proBNP and WBC, were sampled at baseline. Logistic regression analysis adjusted for EuroSCORE-II tested associations between biomarker levels and VARC-III adjudicated endpoints. Model fit was assessed using Akaike's Information Criterion, and likelihood ratio tests compared different prediction models.

**Results:**

Preoperative hemoglobin (OR 0.70; 95% CI: 0.57, 0.87; *p* < 0.001), creatinine (OR 4.09; 95% CI: 1.63, 10.26; *p* = 0.003), high-sensitivity troponin I (OR 1.48; 95% CI: 1.11, 1.95; *p* = 0.007), GOT (OR 2.49; 95% CI: 1.06, 5.86; *p* = 0.036), INR (OR 4.74; 95% CI: 1.32, 17.05; *p* = 0.017), CRP (OR 2.14; 95% CI: 1.53, 3.00; *p* < 0.001), NT-proBNP (OR 2.06; 95% CI: 1.41, 3.00; *p* < 0.001), and WBC (OR 5.82; 95% CI: 1.88, 17.97; *p* = 0.002) were independently associated with 30-day mortality after adjustment for EuroSCORE-II. Models combining biomarkers with EuroSCORE-II outperformed those predicting mortality by EuroSCORE-II or biomarkers alone, as indicated by the lowest Akaike's Information Criterion and likelihood ratio tests.

**Conclusions:**

Combining established risk stratification models with preoperative biomarkers was associated with improved predictive performance for adverse outcomes after aortic valve surgery and may support heart team decision-making when choosing between surgical and transcatheter aortic valve replacement.

## Introduction

According to current guidelines (1), a crucial part of interdisciplinary heart team discussions involves the assessment of anticipated procedural risk and differential allocation between surgical aortic valve replacement (SAVR) and transcatheter aortic valve replacement (TAVR) in patients with relevant aortic valve disease. However, prediction of mortality or postoperative complications after cardiac surgery is challenging. In addition to established risk stratification tools (e.g., EuroSCORE-II (ES-II) or Society of Thoracic Surgeons Score (STS score)), preoperative determination of biomarkers may be helpful to predict postoperative adverse outcomes. To further elucidate this topic, we aimed to investigate the association between preoperative measured circulating biomarkers with postoperative complication rates after aortic valve surgery.

## Material & methods

### Study population

Between March 2018 and May 2022, 492 consecutive patients underwent aortic valve surgery at the University Heart & Vascular Center Hamburg due to severe aortic valve disease according to current guidelines (1) and were included for analysis. Prospectively, 265 patients were included. To increase the analyzed cohort, another 227 patients were added retrospectively. Patients undergoing concomitant procedures such as coronary artery bypass graft, mitral and tricuspid valve surgery were also included. Concomitant aortic root surgery (Bentall's, David's, Yacoub's or Ross' procedures) were excluded, since patients with thoracic aortic surgery have higher risk for peri- and postoperative stroke as shown by Demal et al., with an incidence of 7.0% (2), whereas SAVR patients have an occurrence of stroke of 1.1% in the German Aortic valve registry (3).

A larger and more heterogeneous cohort was chosen to increase the number of total events, thereby allowing the inclusion of more covariates. The ES-II was added as a covariate to balance out the influence of baseline parameters, endocarditis and combined procedures, so that multiple biomarkers could be analyzed. Furthermore, two subgroup analysis were conducted (1): excluding patients with endocarditis and (2): including only patients undergoing isolated SAVR.

This study was conducted in accordance with the Declaration of Helsinki and approved by the Hamburg Ethics Committee (protocol code: PV4304, date of approval: 31 January 2013). Patients either consented to data collection as part of the prospective cohort study (“HARbOR”, NCT 04227002) or ethical approval and informed consent to participate was waived in compliance with the local legislation and institutional requirements (§12 HmbKHG).

### Biomarker analysis

Patients underwent routine preoperative sampling of the following biomarkers: hemoglobin, creatinine, hsTrop-I, glutamate oxaloacetate transaminase (GOT), glutamate pyruvate transaminase (GPT), INR (International normalized ratio), C-reactive protein (CRP), N-terminal Pro-B-Type Natriuretic Peptide (NT-proBNP), and white blood cells (WBC). Peripheral venous blood was drawn less than three days before surgery into pyrogen-free tubes (S-Monovette®, Citrat, EDTA, Lithium-Heparin or Serum-Gel Sarstedt, Nuembrecht, Germany). Detailed protocols of biomarker measurements are found in the methods section of the supplemental material.

### Statistical analysis

Continuous variables are reported as median and Interquartile range (IQR). Binary variables are shown as counts and percentages.

Associations between the outcome parameters 30-day mortality, renal failure, stroke, life-threatening bleeding type 3-4 as well as the combined 30-day VARC-III endpoints early safety and clinical efficacy (dependent variables) with the tested biomarkers (independent variables) were assessed using logistic regression analysis. All biomarkers were log-transformed before analysis. Regression analysis was adjusted for ES-II and INR. Due to a low event rate a Firth correction was performed. Finally, accuracy of three different regression models for prediction of 30-day mortality was compared. In these models, independent variables were: Model 1, ES-II only; Model 2, Biomarkers only; Model 3, a combination of both ES-II and biomarkers. The model fit was compared using Akaike's Information Criterion (AIC). Additionally, likelihood ratio tests (LRT) for nested models were performed to compare the adjusted biomarker model (Model 3) to the univariable ES-II model (Model 1). For non-nested models, LRTs (Model 1 vs. Model 2) are impossible, since two logistic models with different variables cannot be compared using a *p*-value. All statistical analyses were performed using R version 4.2.1 (R Foundation for Statistical Computing). *P*-values <0.05 were considered statistically significant.

## Results

Patient characteristics and biomarker levels are presented in Table 1 and revealed a predominantly male, generally low-risk patient, heterogeneous patient population with regard to the leading indication for surgery. Procedural data are listed in Table 2. Surgical aortic valve replacement was performed in 469 (95.4%) and aortic valve repair in 23 patients (4.6%). Concomitant procedures were performed in 174 patients (35.4%).

**Table 1:** Baseline characteristics.

Baseline characteristics	*n* = 492
Age (years), median (IQR)	67.4 (58.0, 74.3)
Male gender, *n* (%)	341 (69.5)
EuroSCORE-II (%), median (IQR)	3.0 (1.7, 18.3)
Previous sternotomy, *n* (%)	86 (17.5)
Ejection fraction (%), median (IQR)	56.0 (51.0, 60.0)
>50%, *n* (%)	352 (76.4)
31%–50%, *n* (%)	59 (12.8)
21–30%, *n* (%)	29 (6.3)
<21%, *n* (%)	21 (4.6)
Chronic lung disease, *n* (%)	38 (7.7)
Diabetes, *n* (%)	92 (18.7)
Coronary artery disease, *n* (%)	206 (42.0)
Extracardiac arteriopathy, *n* (%)	24 (4.9)
Prior stroke, *n* (%)	50 (10.2)
Coumarin intake, *n* (%)	24 (4.9)
Leading indication for surgery	
Regurgitation, %	19.6
Stenosis, %	51.5
Mixed %	12.9
Endocarditis, %	16.0
Biomarkers	
Hemoglobin (g/dL), median (IQR)	13.6 (12.3, 14.6)
Creatinine (mg/dL), median (IQR)	1.0 (0.8, 1.1)
hsTrop-I (pg/mL), median (IQR)	15.0 (7.0, 39.2)
GOT (U/l), median (IQR)	25.0 (20.0, 32.3)
GPT (U/l), median (IQR)	24.0 (18.0, 34.0)
INR, median (IQR)	1.0 (1.0, 1.1)
CRP (mg/l), median (IQR)	5.0 (4.0, 9.0)
NT-proBNP (ng/l), median (IQR)	675.0 (237.0, 2317.8)
WBC (10^6/L), median (IQR)	7.3 (5.8, 9.0)

CRP, C-reactive protein; GOT, glutamic oxaloacetic transaminase; GPT, glutamate pyruvate transaminase; hsTrop-I, high-sensitivity troponin I; INR, international normalized ratio; IQR, interquartile range; NT-proBNP, N-terminal pro B-type natriuretic peptide; WBC, white blood cells.

**Table 2:** Procedural data.

Procedural data	*n* = 492
Procedural time (minutes), median (IQR)	210.0 (168.0, 260.0)
Cardiopulmonary bypass time (minutes), median (IQR)	110.0 (84.4, 143.0)
Cross-clamp time (minutes), median (IQR)	75.0 (58.0, 101.4)
Aortic valve repair, *n* (%)	23 (4.6)
Aortic valve replacement, *n* (%)	469 (95.4)
Mechanical prosthesis, *n* (%)	10 (2.1)
Biological prosthesis, *n* (%)	456 (97.9)
Prosthesis, *n* (%)	
Edwards Perimount/Magna Ease	239 (51.3)
Edwards Inspiris Resilia	69 (14.8)
Medtronic Hancock	60 (12.9)
Medtronic Avalus	52 (11.1)
LivaNova Perceval	31 (6.7)
On-X	6 (1.3)
SJM Standard/Trifecta	7 (1.5)
Corlife Homograft	2 (0.4)
Prosthesis label size (mm), median (IQR)	25.0 (23.0, 25.0)
Minimally invasive access, *n* (%)	167 (34.2)
Root enlargement, *n* (%)	1 (0.2)
Concomitant procedures, *n* (%)	174 (35.4)
Coronary artery bypass grafting	102 (20.8)
Mitral valve surgery	83 (16.9)
Tricuspid valve surgery	30 (6.1)

IQR, interquartile range.

Procedural and 30-day outcome parameters are listed in Table 3. Overall 30-day mortality, disabling stroke and myocardial infarction were 3.5% (*n* = 17), 2.2% (*n* = 11) and 1.2% (*n* = 6) respectively. In 6.3% (*n* = 31) of the patients, bleeding type 3 or 4 was documented. Combined VARC-III endpoints early safety and clinical efficacy were reached in 73.9% (*n* = 362) and 93.6% (*n* = 457) of patients, respectively. Device success was present in all patients except cases with early mortality (95.7%).

**Table 3:** Procedural and 30-day outcome.

Early outcome parameters	*n* = 492
30-day mortality, *n* (%)	17 (3.5)
Acute kidney injury, *n* (%)	
Stage I	56 (11.4)
Stage II	6 (1.2)
Stage III	10 (2.0)
Stage IV (dialysis)	43 (8.8)
Disabling stroke, *n* (%)	11 (2.2)
Myocardial infarction, *n* (%)	6 (1.2)
Bleeding type 3 or 4, *n* (%)	31 (6.3)
Permanent pacemaker implantation, *n* (%)	42 (8.7)
Mean transvalvular gradient (mmHg), median (IQR)	11.0 (8.0, 14.0)
Moderate or severe paravalvular leakage, *n* (%)	4 (0.9)
Combined 30-day VARC-III endpoints	
Device success, *n* (%)	468 (95.7)
Early safety, *n* (%)	362 (73.9)
Clinical efficacy, *n* (%)	457 (93.6)

IQR, interquartile range.

**Figure 1:**
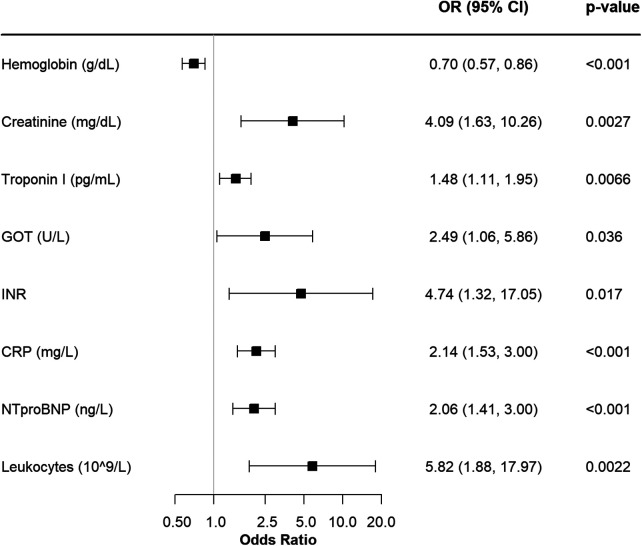
Forest plot of significant biomarkers predicting 30-day-mortality identified by adjusted logistic regression analysis. CRP, C-reactive protein; GOT, glutamic-oxalacetic transaminase; INR, international normalized ratio; NT-proBNP, N-terminal pro B-type natriuretic peptide.

**Figure 2:**
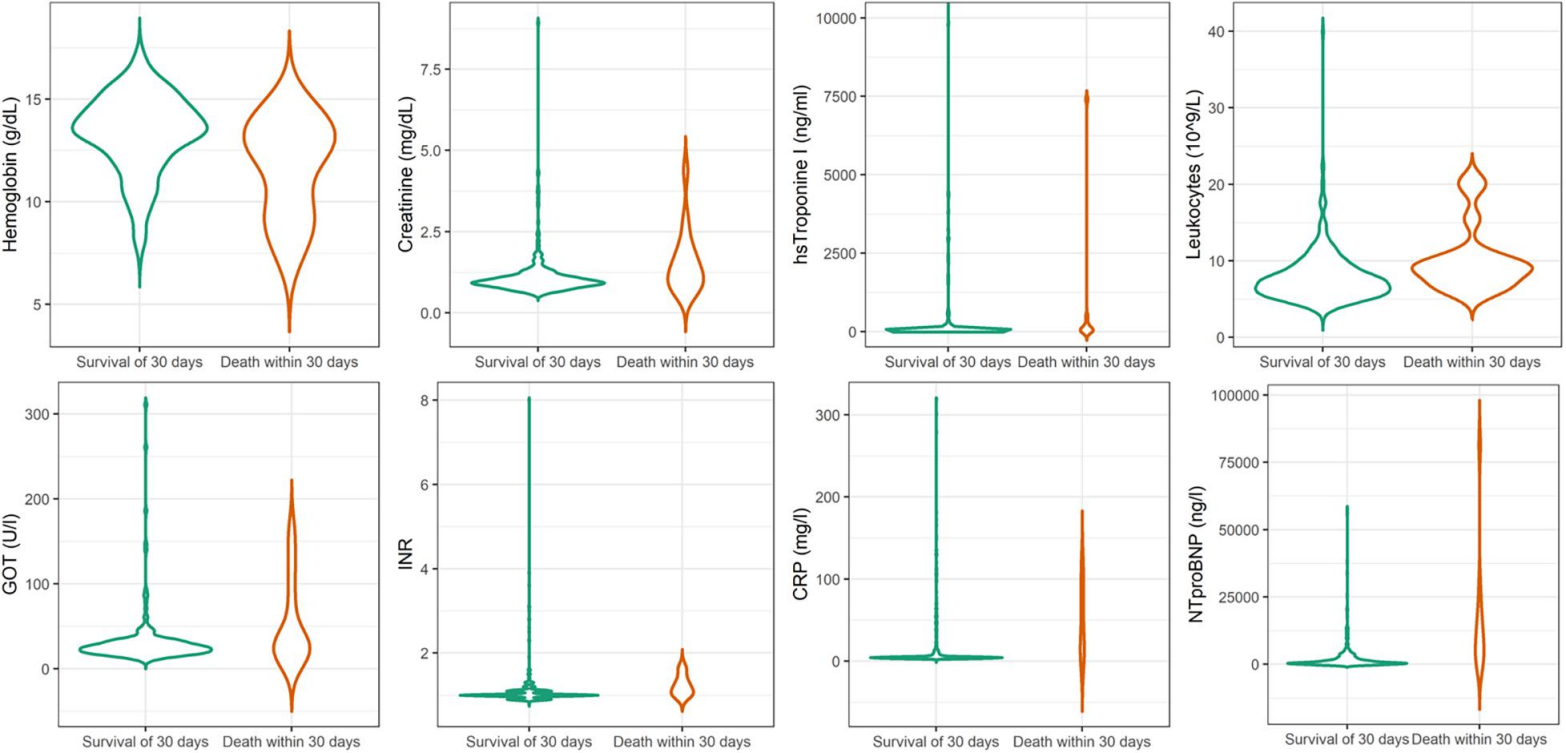
Distribution of significant biomarkers levels predicting mortality in 30-day survivors vs. non-survivors. Biomarker levels are shown as violin plots. CRP, C-reactive protein; GOT, glutamic-oxalacetic transaminase; INR, international normalized ratio; NT-proBNP, N-terminal pro B-type natriuretic peptide.

Associations of the biomarkers with 30-day mortality and other postoperative outcome parameters are shown in Figure 1 and Table 4. Preoperative hemoglobin (OR 0.70; 95% CI: 0.57, 0.87; *p* < 0.001), creatinine (OR 4.09; 95% CI 1.63, 10.26; *p* = 0.003), hsTrop-I (OR 1.48; 95% CI 1.11, 1.95; *p* = 0.007), GOT (OR 2.49; 95% CI 1.06, 5.86; *p* = 0.036), INR (OR 4.74; 95% CI 1.32, 17.05; *p* = 0.020), CRP (OR 2.14; 95% CI 1.53, 3.00; *p* < 0.001), NT-proBNP (OR 2.06; 95% CI 1.41, 3.00; *p* < 0.001), and WBC (OR 5.82; 95% CI: 1.88, 17.97; *p* = 0.002) were all significant and independent predictors (adjustment for ES-II) for 30-day mortality. After adjustment for Coumarin intake, INR could not be confirmed as an independent predictor for 30-day mortality (OR 4.22; 95% CI 0.47, 37.81; *p* = 0.200). In Figure 2 the distribution of significant biomarker levels in 30-day-survivors vs. non-survivors are shown as violin plots.

**Table 4:** Regression analysis adjusted by EuroSCORE II.

Biomarker	30-day mortality		Renal failure		Stroke		Life-threatening bleeding		Early safety		Clinical efficacy	
	OR (95% CI)	*p*-value	OR (95% CI)	*p*-value	OR (95% CI)	*p*-value	OR (95% CI)	*p*-value	OR (95% CI)	*p*-value	OR (95% CI)	*p*-value
Hemoglobin (g/dL)	0.70 (0.57, 0.87)	<0.001	0.69 (0.62, 0.77)	<0.001	0.95 (0.72, 1.26)	0.730	0.80 (0.68, 0.94)	0.007	1.38 (1.24, 1.53)	<0.001	1.27 (1.08, 1.50)	0.004
Creatinine (mg/dL)	4.09 (1.63, 10.26)	0.003	11.84 (5.74, 24.42)	<0.001	1.42 (0.32, 6.29)	0.650	2.92 (1.31, 6.52)	0.009	0.22 (0.12, 0.41)	<0.001	0.35 (0.16, 0.79)	0.011
hsTrop-I (pg/mL)	1.48 (1.11, 1.95)	0.007	1.38 (1.16, 1.63)	<0.001	1.22 (0.82, 1.84)	0.330	1.26 (0.95, 1.66)	0.110	0.83 (0.71, 0.97)	0.020	0.69 (0.52, 0.90)	0.006
GOT (U/l)	2.49 (1.06, 5.86)	0.036	2.42 (1.36, 4.32)	0.003	2.33 (0.78, 6.90)	0.130	1.35 (0.55, 3.32)	0.510	0.73 (0.42, 1.24)	0.240	0.49 (0.22, 1.10)	0.085
GPT (U/l)	1.37 (0.60, 3.14)	0.460	1.01 (0.69, 1.48)	0.970	1.01 (0.36, 2.83)	0.980	0.96 (0.49, 1.88)	0.910	1.16 (0.79, 1.68)	0.450	1.05 (0.53, 2.07)	0.880
INR	4.74 (1.32, 17.05)	0.017	4.21 (1.68, 10.58)	0.002	0.94 (0.06, 15.60)	0.970	2.32 (0.68, 7.89)	0.180	0.25 (0.10, 0.61)	0.003	0.30 (0.09, 0.97)	0.044
INR (adjusted for Coumarin intake)	4.22 (0.47, 37.81)	0.200	4.45 (1.15, 17.29)	0.031	1.06 (0.03, 35.47)	0.970	1.27 (0.20, 8.24)	0.800	0.26 (0.07, 0.98)	0.047	0.42 (0.07, 2.62)	0.350
CRP (mg/l)	2.14 (1.53, 3.00)	<0.001	1.71 (1.43, 2.06)	<0.001	0.85 (0.46, 1.57)	0.600	1.33 (1.00, 1.76)	0.049	0.59 (0.49, 0.71)	<0.001	0.63 (0.48, 0.82)	<0.001
NT-proBNP (ng/l)	2.06 (1.41, 3.00)	<0.001	1.72 (1.45, 2.04)	<0.001	1.05 (0.72, 1.53)	0.790	0.75 (0.57, 1.00)	0.051	0.73 (0.62, 0.84)	<0.001	0.69 (0.53, 0.90)	0.006
WBC (10^9/L)	5.82 (1.88, 17.97)	0.002	3.21 (1.74, 5.91)	<0.001	1.44 (0.30, 6.82)	0.650	1.30 (0.47, 3.54)	0.610	0.34 (0.19, 0.62)	<0.001	0.29 (0.12, 0.72)	0.008

Logistic regression analysis results showing associations of biomarkers with 30-day postoperative outcome parameters. Parameters are all adjusted for the ES-II and Firth-corrected due to low event rates. INR is additionally adjusted for Coumarin intake. CI, confidence interval; CRP, C-reactive protein; GOT, glutamic oxaloacetic transaminase; GPT, glutamate pyruvate transaminase; INR, international normalized ratio; NT-proBNP, N-terminal pro B-type natriuretic peptide; WBC, white blood cells.

Renal failure was predicted by hemoglobin (OR 0.69; 95% CI 0.62, 0.77; *p* < 0.001), creatinine (OR 11.84; 95% CI 5.74, 24.42; *p* < 0.001), hsTrop-I (OR 1.38; 95% CI 1.16, 1.63; *p* < 0.001), GOT (OR 2.42; 95% CI 1.36, 4.32; *p* = 0.003), INR (OR 4.21; 95% CI 1.68, 10.58; *p* = 0.002), INR adjusted for Coumarin intake (OR 4.45; 95% CI 1.15, 17.29; *p* = 0.031), CRP (OR 1.71; 95% CI 1.43, 2.06; *p* < 0.001), NT-proBNP (OR 1.72; 95% CI 1.45, 2.04; *p* < 0.001), and WBC (OR 3.21; 95% CI: 1.74, 5.91; *p* < 001).

Independent predictors for life-threatening bleeding (VARC bleeding type 3/4) were hemoglobin (OR 0.80; 95% CI 0.68, 0.94; *p* = 0.007), creatinine (OR 2.92; 95% CI 1.31, 6.52; *p* = 0.009), and CRP (OR 1.33; 95% CI 1.00, 1.76; *p* = 0.049).

None of the tested biomarkers independently correlated postoperative stroke occurrence.

The combined VARC-III endpoint early safety was predicted by hemoglobin (OR 1.38; 95% CI 1.24, 1.53; *p* < 0.001), creatinine (OR 0.22, 95% CI 0.12, 0.41; *p* < 0.001), hsTrop-I (OR 0.83, 95% CI 0.71, 0.97; *p* = 0.020), INR (OR 0.25; 95% CI 0.10, 0.61; *p* = 0.003), INR adjusted for Coumarin intake (OR 0.26; 95% CI 0.07, 0.98, *p* = 0.047), CRP (OR 0.59; 95% CI 0.49, 0.71; *p* < 0.001), NT-proBNP (OR 0.73; 95% CI 0.62, 0.84; *p* < 0.001), and WBC (OR 0.34; 95% CI: 0.19, 0.62; *p* < 0.001).

Independent predictors for the combined VARC-III endpoint clinical efficacy were hemoglobin (OR 1.27; 95% CI 1.08, 1.50; *p* = 0.004), creatinine (OR 0.35; 95% CI 0.16, 0.79; *p* = 0.011), hsTrop-I (OR 0.69; 95% CI 0.52, 0.90; *p* = 0.006), INR (OR 0.30; 95% CI 0.09, 0.97; *p* = 0.044), CRP (OR 0.63; 95% CI 0.48, 0.82; *p* < 0.001), NT-proBNP (OR 0.69; 95% CI 0.53, 0.90; *p* = 0.006), and WBC (OR 0.29; 95% CI: 0.12, 0.72; *p* = 0.008).

**Figure 3:**
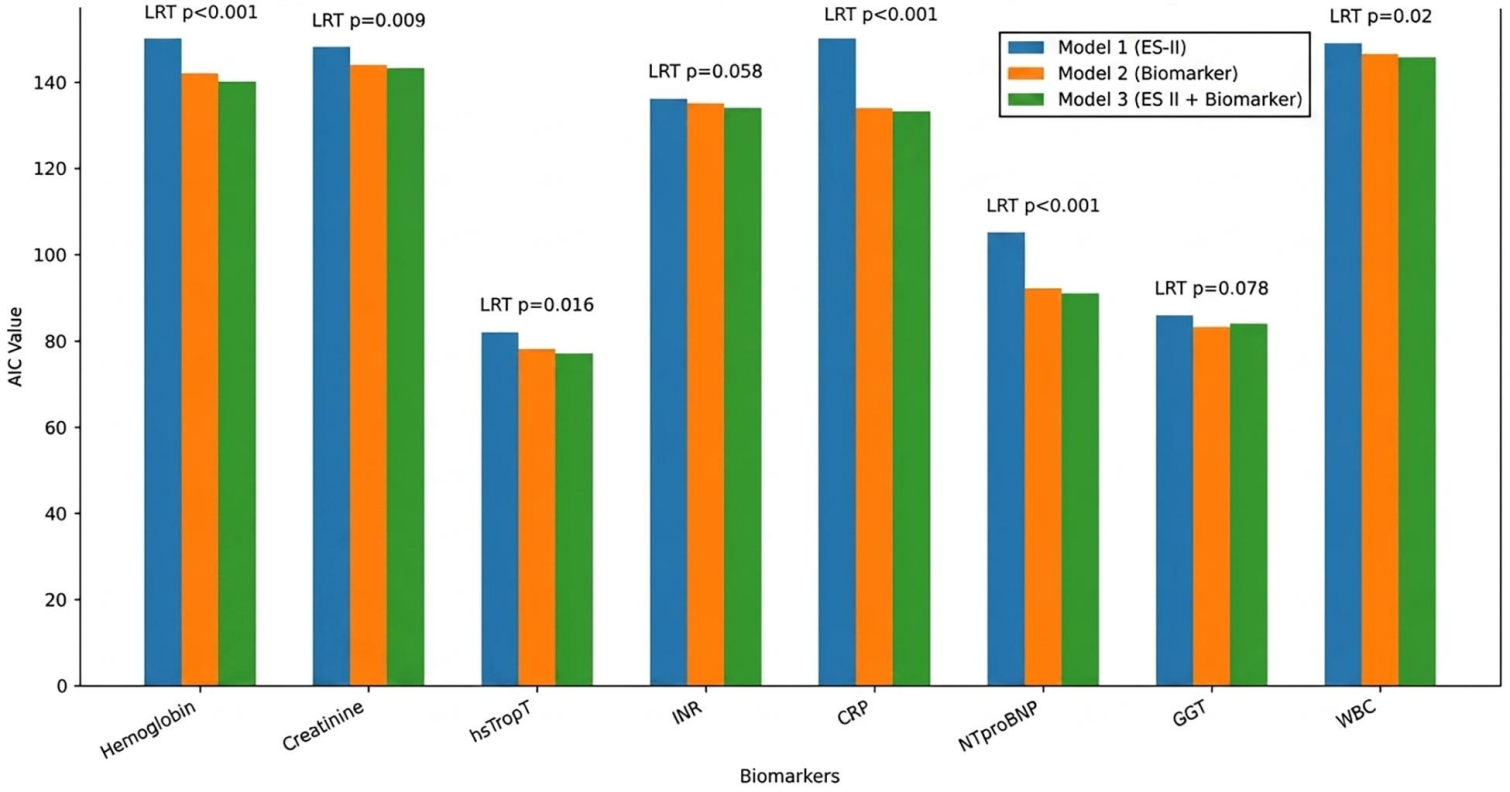
Comparisons of the different models predicting 30-day mortality. The accuracy of each model is indicated by the AIC. The smaller the AIC value, the better the model fit. Model 1 and model 3 are compared using likelihood ratio tests. If the *p*-value of the LRT is ≤ 0.05 for the model 1 vs. model 3 comparison, then model 3 has the higher prediction accuracy. AIC, akaike's information criterion; CRP, C-reactive protein; GOT, glutamic-oxaloacetic transaminase; INR, International normalized ratio; LRT, likelihood ratio test.

Comparisons of the different models predicting 30-day mortality are shown in Figure 3. Model 1 is characterized by the prediction using ES-II only, Model 2 by biomarkers only, and Model 3 by the combination of both ES-II and biomarkers.

Here, the combination of the ES-II and biomarkers predicts 30-day mortality best as indicated by lowest AIC value for hemoglobin (AIC 140.4), creatinine (AIC 144), hsTrop-I (AIC 78.5), INR (AIC 133.8), CRP (AIC 132), NT-proBNP (AIC 92) and WBC (AIC 145.6). Since every biomarker is its own model, the AIC cannot be compared between different biomarkers.

Accordingly, the LRT also suggests that using the combination of ES-II and biomarkers results in a better prediction of 30-day mortality than the use of ES-II alone if hemoglobin (*p* = 0.001), creatinine (*p* = 0.009), hsTrop-I (*p* = 0.016), CRP (*p* < 0.001), NT-proBNP (*p* < 0.001) or WBC (*p* = 0.020) are used as predicting biomarkers.

**Figure 4:**
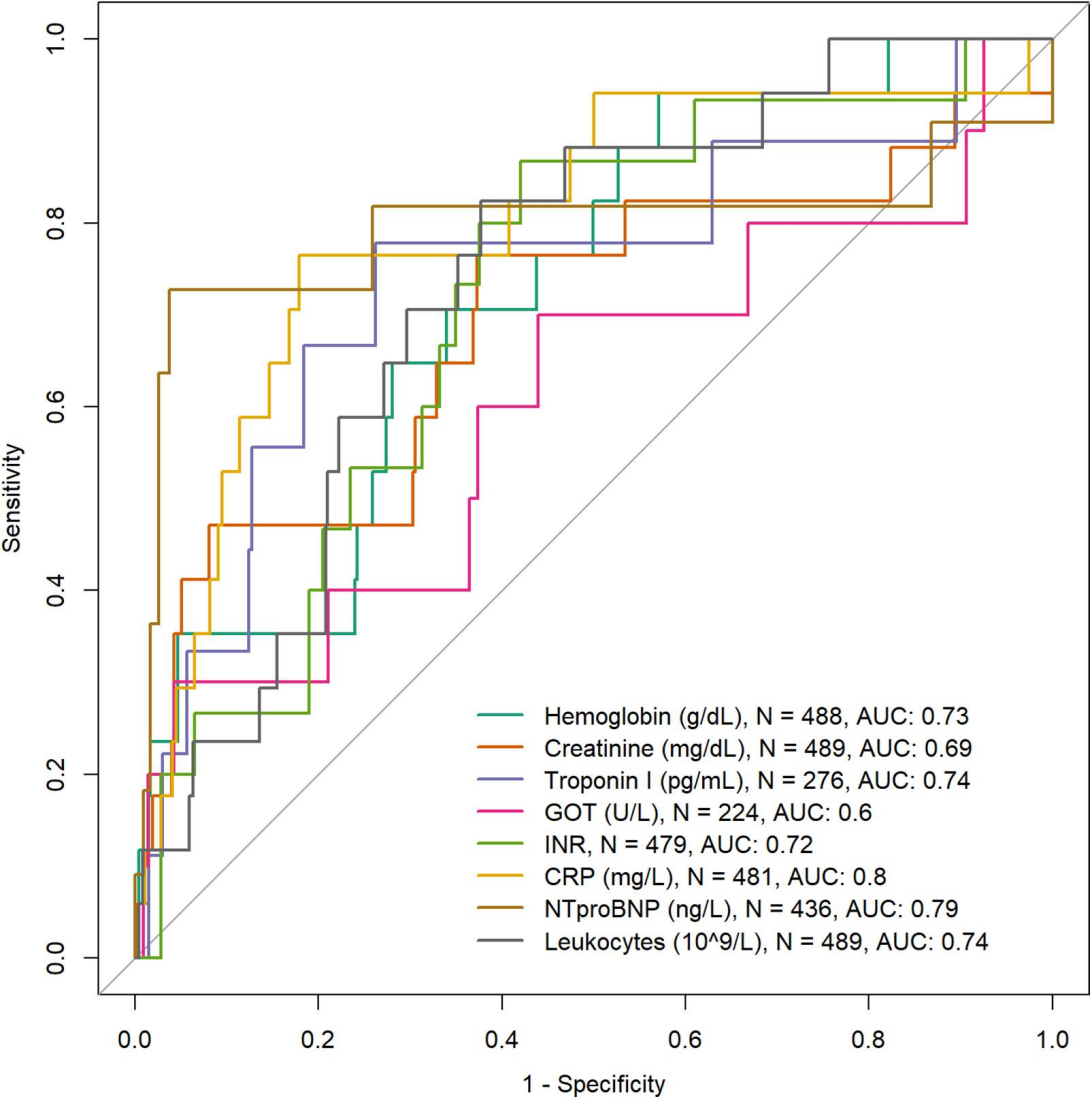
Receiver operating characteristic curves of adjusted biomarkers predicting 30-day mortality. AUC, area under the curve; CRP, C-reactive protein; INR, international normalized ratio; NT-proBNP, N-terminal pro B-type natriuretic peptide.

 Figure 4 shows receiver operating characteristic (ROC) curves of significant biomarkers adjusted by ES-II predicting 30-day mortality. Predictive performance was good using hemoglobin, hsTrop-I, INR, NT-proBNP, and WBC with area under the curve (AUC) values between 0.7 and 0.8. CRP performs best with the highest AUC of 0.8.

As a sensitivity analysis, we examined the subcohort of patients undergoing isolated SAVR (see Supplementary Tables S1–S4). In this group, 30-day mortality was predicted by preoperative levels of hemoglobin (OR 0.55; 95% CI: 0.34, 0.88; *p* = 0.013) and CRP (OR 2.64; 95% CI: 1.43, 4.86; *p* = 0.002). INR (OR 5.23; 95% CI: 0.99, 27.62; *p* = 0.051) and hsTrop-I (OR 1.49; 95% CI: 0.99, 2.24; *p* = 0.053) also showed a trend towards predicting 30-day mortality.

Furthermore, we examined the cohort of patients without endocarditis (see Supplementary Tables S5–S8). Here, 30-day mortality was predicted by creatinine (OR 4.84; 95% CI: 1.20, 19.50; *p* = 0.027), NTproBNP (OR 2.23; 95% CI: 1.30, 3.85; *p* = 0.003). A trend predicting 30-day mortality was shown by CRP (OR 1.94; 95% CI: 0.99, 3.80; *p* = 0.055).

## Discussion

Our analysis shows a strong association between preoperatively tested routine blood biomarkers and early outcome after SAVR. The analysis indicates that multiple biomarkers combined with ES-II may provide better prediction in SAVR patients than the ES-II alone. The ES-II is widely used in the preoperative risk assessment in cardiac surgery. It shows a high prediction accuracy as demonstrated by prior studies (4, 5). Nonetheless, the analysis of Chalmers et al. (6) showed that the ES-II did not improve on the original EuroSCORE in risk prediction in isolated SAVR procedures. Additionally, multiple studies (7, 8) indicate that the ES-II is not a reliable predictor in high-risk patients. Accordingly, Grant et al. (5) and Borde et al. (9) demonstrated an over-estimation of mortality in high-risk patients by ES-II.

The use of biomarkers in the prediction of the postoperative outcome after cardiac surgery has been proposed by several prior studies (10). Here, especially creatinine (11) and NT-proBNP (12) have shown reasonable accuracy. Additionaly, Salama et al. demonstrated that high-sensitivity troponin T has prognostic value for major adverse cardiovascular events in patients with severe aortic stenosis and preserved left ventricular ejection fraction undergoing SAVR (13). Prior to our analysis, Spampinato et al. investigated blood biomarkers predicting postoperative outcomes after SAVR for aortic stenosis (14). Here, combining the biomarkers NT-proBNP, hsTrop-T, and CRP with the EuroSCORE results in a better prediction profile than using EuroSCORE alone. However, their study used the original EuroSCORE which includes data of 19,030 patients, from which a high rate underwent coronary artery bypass grafting and only 5,671 patients received valve surgery (15). In our study, the more accurate ES-II was used, which is based on a larger cohort of 22,381 patients, of whom 10,353 of the patients underwent valve surgery (16).

Although strong correlations were found between several tested blood biomarkers and outcome parameters after SAVR, the causal connection between biomarker levels and occurring complications was not investigated. Whereas the correlations between bleeding events and preoperative INR as well as between creatinine and renal failure appear self-explanatory, other associations are more difficult to understand. However, high levels of NT-proBNP and hsTrop-I may indicate preoperative heart failure and/or cardiac ischemia impairing the periprocedural outcome. Elevated WBC and/or CRP levels may either indicate perioperative inflammation and/or infections or reflect an increased cardiovascular risk and are therefore associated with impaired postprocedural outcome. Noteworthy, both WBC and CRP demonstrate additional predictive performance beyond the information provided by the presence or absence of endocarditis. This is because the predictive models for CRP are adjusted for ES-II, within which endocarditis is included as a binary parameter. Finally, low hemoglobin levels may be caused by malnutrition or chronic diseases like malignant tumors or chronic inflammatory syndromes which may elevate the periprocedural risk.

The main limitation of this study is the single center design. In addition anemia-related parameters reflecting iron stores were not assessed. We therefore propose prospective multicenter analyses in future studies on risk predicting blood biomarkers in cardiac surgery.

In the context of preconditioning, the identified biomarkers reflect potentially modifiable factors, indicating that targeted optimization before surgery could be beneficial. Structured patient blood management and assessment of systemic inflammation may therefore represent important elements of prehabilitation and should be considered within Enhanced Recovery After Surgery (ERAS) programs.

## Conclusion

This analysis identified the biomarkers hemoglobin, hsTrop-I, INR, NT-proBNP, WBC and CRP that predict postprocedural clinical endpoints after aortic valve surgery including 30-day mortality. We cautiously assume that these biomarkers may indicate baseline cardiac and/or end-organ injury. The prediction model combining biomarkers and ES-II showed a superior accuracy when compared to the models using ES-II or biomarkers alone. After validation of the proposed biomarkers in larger multicenter cohorts, their use may simplify heart team discussions and decision-making between SAVR and TAVR in patients with aortic valve diseases.

## Data Availability

The original contributions presented in the study are included in the article/Supplementary Material, further inquiries can be directed to the corresponding author.
