# Supplementary material for: Circulating biomarkers improve prediction of postoperative outcome after aortic valve surgery
Source: Front Cardiovasc Med. 2026 Jun 2;13:1813821. doi: 10.3389/fcvm.2026.1813821 (PMC13270467; doi:10.3389/fcvm.2026.1813821)
Supplement: Supplementary file 1 [file Datasheet1.docx]

**Supplemental Material**

**Circulating biomarkers for prediction of postoperative outcome after aortic valve surgery**

Till Joscha Demal MD^1,4#^; Jenny Bialczak MD^1#^; Alina Goßling MSc²; Francisco Miguel Ojeda PhD²; Oliver D. Bhadra MD^1^; Björn Sill MD^1^, Johannes Petersen MD^1^, Sebastian Ludwig MD²; David Grundmann MD²; Lisa Voigtländer MD²; Lara Waldschmidt MD²; Laura Hannen MD^2^; Niklas Schofer MD²; Stefan Blankenberg MD²^,4^; Paulus Kirchhof MD²^,4^; Thomas Renné MD^3^; Lenard Conradi MD^1^; Hermann Reichenspurner MD, PhD^1,4^; Evaldas Girdauskas MD^1,4^; Andreas Schaefer MD, MHBA^1^

*1 Department of Cardiovascular Surgery, University Heart and Vascular Center Hamburg, Hamburg, Germany
2 Department of Cardiology, University Heart and Vascular Center Hamburg, Hamburg, Germany*

*3 Department of Clinical Chemistry and Laboratory Medicine, University Medical Center Hamburg-Eppendorf, Hamburg, Germany*

*4 German Center for Cardiovascular Research, partner site Hamburg/Kiel/Lübeck, Germany*

# both authors contributed equally

Corresponding author: Jenny Bialczak

University Heart and Vascular Center Hamburg

Department of Cardiovascular Surgery

Martinistraße 52

D-20246 Hamburg

Germany

e-mail: j.bialczak@uke.de

Phone: +49 (0) 40 7410 - 52440

Fax: +49 (0) 40-7410 - 57926

**MATERIAL & METHODS**

Detailed protocols of biomarker analyses

Patients underwent routine preoperative sampling of the following biomarkers: hemoglobin, creatinine, high-sensitive troponin I (hsTrop-I), glutamate oxaloacetate transaminase (GOT), glutamate pyruvate transaminase (GPT), INR (International normalized ratio), c-reactive Protein (CRP), and N-Terminal Pro-B-Type Natriuretic Peptide (NT-proBNP). Peripheral venous blood was drawn less than three days before surgery into pyrogen-free tubes (S-Monovette®, Citrat, EDTA, Lithium-Heparin or Serum-Gel Sarstedt, Nuembrecht, Germany).

Hemoglobin and WBC concentrations were measured by photometry on Siemens Advia 2120 high-volume hematology system (Siemens Healthineers^TM^, Erlangen, Germany). Creatinine, INR, GOT and GPT levels were calculated photometrically on a clinical chemistry modular analyzer (Atellica Solution CH930, Siemens Healthineers^TM^, Erlangen, Germany). Levels of CRP were determined using a particle-enhance turbidimetric immunoassay on a clinical chemistry modular analyzer (Atellica Solution CH930, Siemens Healthineers^TM^, Erlangen, Germany). Concentrations of hsTrop-I and NT-proBNP were measured using the chemiluminescent immunoassay method on a Siemens Atellica Solution IM 1600 analyzer (Siemens Healthineers™, Erlangen, Germany).

**SUBSET ANALYSIS 1: ISOLATED SURGICAL AORTIC VALVE REPLACEMENT**

**Table S1**

Baseline characteristics

|  | (n=294) |
| --- | --- |
| Age (years), median (IQR) | 66.2 (57.6, 72.9) |
| Male gender, n (%) | 192 (65.5) |
| ES-II (%), median (IQR) | 2.4 (1.5, 55.8) |
| Previous sternotomy, n (%) | 52 (17.7) |
| Ejection fraction (%), median (IQR) | 56.0 (51.2, 61.0) |
| >50%, n (%) | 227 (80.2) |
| 31–50%, n (%) | 28 (9.9) |
| 21-30%, n (%) | 16 (5.7) |
| <21%, n (%) | 12 (4.2) |
| Chronic lung disease, n (%) | 18 (6.1) |
| Diabetes, n (%) | 46 (15.6) |
| Coronary artery disease, n (%) | 78 (26.6) |
| Extracardiac artheropathy, n (%) | 7 (2.4) |
| Prior stroke, n (%) | 30 (10.2) |
| Coumarin intake, n (%) | 13 (4.4) |
| Leading indication for surgery |  |
| Regurgitation, % | 17.3 |
| Stenosis, % | 55.7 |
| Mixed, % | 13.3 |
| Endocarditis, % | 13.7 |
| Biomarkers |  |
| Hemoglobin (g/dL), median (IQR) | 13.6 (12.5, 14.6) |
| Creatinine (mg/dL), median (IQR) | 0.9 (0.8, 1.1) |
| hsTrop-I (pg/ml), median (IQR) | 13.0 (6.0, 32.3) |
| GOT (U/l), median (IQR) | 25.0 (20.0, 32.0) |
| GPT (U/l), median (IQR) | 25.0 (18.0, 34.3) |
| INR, median (IQR) | 1.0 (1.0, 1.1) |
| C-reactive protein (CRP) (mg/l), median (IQR) | 4.5 (4.0, 7.0) |
| NTproBNP (ng/l), median (IQR) | 620.0 (226.8, 1919.7) |

EuroSCORE: European System for Cardiac Operative Risk Evaluation, GOT: Glutamic Oxaloacetic Transaminase, GPT: Glutamate Pyruvate Transaminase, INR: International Normalized Ratio, IQR: Interquartile range, NTproBNP: N-terminal pro B-type natriuretic peptide

**Table S2**

Procedural data

|  | (n=294) |
| --- | --- |
| Procedural time (minutes), mean (IQR) | 186.0 (155.0, 224.0) |
| CPB time (minutes), mean (IQR) | 95.0 (78.0, 116.0) |
| Cross-clamp time (minutes), mean (IQR) | 65.0 (52.5, 82.3) |
| Aortic valve replacement: |  |
| Mechanical prosthesis, n (%) | 7 (2.4) |
| Biological prosthesis, n (%) | 287 (97.6) |
| Prosthesis, n (%) |  |
| Edwards Perimount / Magna Ease | 239 (51.3) |
| Medtronic Hancock (II) | 60 (12.9) |
| Edwards Inspiris Resilia | 69 (14.8) |
| Medtronic Avalus | 52 (11.1) |
| LivaNova Perceval | 31 (6.7) |
| On-X | 6 (1.3) |
| SJM Standard | 4 (0.9) |
| SJM Trifecta | 3 (0.6) |
| Corlife Homograft | 2 (0.004) |
| Prosthesis label size (mm), mean (IQR) | 25.0 (23.0, 25.0) |
| Minimally invasive access, n (%) | 146 (49.8) |
|  |  |

CPB: cardiopulmonary bypass, IQR: Interquartile range.

**Table S3**

Procedural and 30-day outcome

|  | (n=294) |
| --- | --- |
| 30-day mortality, n (%) | 6 (2.0) |
| Acute kidney injury, n (%) |  |
| Stage I | 23 (7.8) |
| Stage II | 1 (0.3) |
| Stage III | 4 (1.4) |
| Stage IV (dialysis) | 12 (4.1) |
| Disabling stroke, n (%) | 6 (2) |
| Myocardial infarction, n (%) | 2 (0,7) |
| Bleeding type 3 or 4, n (%) | 17 (5.9) |
| Permanent pacemaker implantation, n (%) | 20 (6.9) |
| Mean transvalvular gradient (mmHg), median (IQR) | 11.0 (8.0, 15.0) |
| Moderate or severe PVL, n (%) | 1 (0.4) |
| Combined 30-day VARC-III endpoints |  |
| Device success, n (%) | 288 (98.0) |
| Early safety, n (%) | 239 (81.3) |
| Clinical efficacy, n (%) | 284 (96.6) |

IQR: Interquartile range, PVL: paravalvular leakage.

**Table S4**

Regression analysis adjusted by ES-II

|  | OR (95% CI) | p-value |
| --- | --- | --- |
| Hemoglobin (g/dL) | 0.55 (0.34, 0.88) | **0.013** |
| Creatinine (mg/dL) | 3.15 (0.30, 33.54) | 0.340 |
| hsTrop I (pg/ml) | 1.49 (0.99, 2.24) | 0.053 |
| GOT (U/l) | 1.54 (0.30, 7.96) | 0.610 |
| GPT (U/l) | 0.24 (0.03, 1.88) | 0.180 |
| INR | 5.23 (0.99, 27.62) | 0.051 |
| INR (adjusted for Coumarin intake) | 20547.18 (89.31, 4726964.15) | **<0.001** |
| CRP (mg/l) | 2.64 (1.43, 4.86) | **0.002** |
| NTproBNP (ng/l) | 0.64 (0.24, 1.73) | 0.380 |
|  |  |  |

Logistic regression analysis results showing the associations of biomarkers with 30-day mortality. Parameters are all adjusted for the ES-II and firth corrected due to low event rates. The model based on INR is additionally adjusted for Coumarin intake. CI: Confidence interval, CRP: C-reactive protein, EuroSCORE: European System for Cardiac Operative Risk Evaluation, GOT: Glutamic Oxaloacetic Transaminase, GPT: Glutamate Pyruvate Transaminase, INR: International Normalized Ratio, NTproBNP: N-terminal pro B-type natriuretic peptide.

**SUBSET ANALYSIS 2: EXCLUDING PATIENTS WITH ENDOCARDITIS**

**Table S5**

Baseline characteristics

|  | n=404 | |
| --- | --- | --- |
| Age (years), median (IQR) | 67.3 (57.8, 74.2) | |
| Male gender, n (%) | 274 (67.8) | |
| ES-II (%), median (IQR) | 2.8 (1.6, 50.2) | |
| Previous sternotomy, n (%) | 63 (15.6) | |
| Ejection fraction (%), median (IQR) | 56.0 (51.0, 60.6) | |
| >50%, n (%) | 51 (13.1) | |
| 31–50%, n (%) | 301 (77.2) | |
| 21-30%, n (%) | 20 (5.1) | |
| <21%, n (%) | 18 (4.6) | |
| Chronic lung disease, n (%) | 26 (6.4) | |
| Diabetes, n (%) | 72 (17.8) | |
| Coronary artery disease, n (%) | 178 (44.3) | |
| Extracardiac artheropathy, n (%) | 19 (4.7) | |
| Prior stroke, n (%) | 37 (9.2) | |
| Coumarin intake, n (%) | 15 (3.7) | |
| Leading indication for surgery |  | |
| Regurgitation, % | 23.5 | |
| Stenosis, % | 58.7 | |
| Mixed, % | 17.8 | |
| Endocarditis, % | 0 | |
| Biomarkers | |  |
| Hemoglobin (g/dL), median (IQR) | 13.8 (12.9, 14.8) | |
| Creatinine (mg/dL), median (IQR) | 1.0 (0.8, 1.1) | |
| hsTrop-I (pg/ml), median (IQR) | 13.5 (6.0, 33.6) | |
| GOT (U/l), median (IQR) | 24.0 (19.0, 32.0) | |
| GPT (U/l), median (IQR) | 24.0 (18.0, 33.3) | |
| INR, median (IQR) | 1.0 (1.0, 1.1) | |
| C-reactive protein (CRP) (mg/l), median (IQR) | 4.0 (4.0, 5.0) | |
| NTproBNP (ng/l), median (IQR) | 559.0 (211.0, 1761.7) | |
| WBC (10^9/L), median (IQR) | 7.1 (5.8, 8.5) | |

EuroSCORE: European System for Cardiac Operative Risk Evaluation, GOT: Glutamic Oxaloacetic Transaminase, GPT: Glutamate Pyruvate Transaminase, INR: International Normalized Ratio, IQR: Interquartile range, NTproBNP: N-terminal pro B-type natriuretic peptide, WBC: white blood cells

**Table S6**

Procedural data

|  | n=404 |
| --- | --- |
| Procedural time (minutes), mean (IQR) | 205.5 (168.0, 254.6) |
| CPB time (minutes), mean (IQR) | 109.5 (85.0, 139.9) |
| Cross-clamp time (minutes), mean (IQR) | 74.4 (59.0, 99.0) |
| Aortic valve replacement: | 373 (94.7) |
| Mechanical prosthesis, n (%) | 7 (1.8) |
| Biological prosthesis, n (%) | 372 (98.2) |
| Prosthesis, n (%) |  |
| Edwards Perimount / Magna Ease | 184 (48.6) |
| Medtronic Hancock (II) | 43 (11.3) |
| Edwards Inspiris Resilia | 65 (17.2) |
| Medtronic Avalus | 51 (13.5) |
| LivaNova Perceval | 27 (7.1) |
| On-X | 4 (1.1) |
| SJM Standard | 3 (0.8) |
| SJM Trifecta | 2 (0.4) |
| Corlife Homograft | 2 (0.004) |
| Prosthesis label size (mm), mean (IQR) | 25.0 (23.0, 25.0) |
| Minimally invasive access, n (%) | 158 (39.4) |
|  |  |

CPB: cardiopulmonary bypass, IQR: Interquartile range.

**Table S7**

Procedural and 30-day outcome

|  | n=404 |
| --- | --- |
| 30-day mortality, n (%) | 9 (2.2) |
| Acute kidney injury, n (%) |  |
| Stage I | 43 (10.7) |
| Stage II | 4 (1.0) |
| Stage III | 6 (1.5) |
| Stage IV (dialysis) | 26 (6.5) |
| Disabling stroke, n (%) | 10 (2.5) |
| Myocardial infarction, n (%) | 6 (1.5) |
| Bleeding type 3 or 4, n (%) | 22 (5.5) |
| Permanent pacemaker implantation, n (%) | 29 (7.3) |
| Mean transvalvular gradient (mmHg), median (IQR) | 11.0 (8.0, 14.0) |
| Moderate or severe PVL, n (%) | 3 (0.8) |
| Combined 30-day VARC-III endpoints |  |
| Device success, n (%) | 392 (97.5) |
| Early safety, n (%) | 313 (77.7) |
| Clinical efficacy, n (%) | 382 (95.3) |

IQR: Interquartile range, PVL: paravalvular leakage.

**Table S8**

Regression analysis adjusted by ES-II

|  | OR (95% CI) | p-value |
| --- | --- | --- |
| Hemoglobin (g/dL) | 0.93 (0.61, 1.40) | 0.720 |
| Creatinine (mg/dL) | 4.84 (1.20, 19.50) | **0.027** |
| hsTrop I (pg/ml) | 1.23 (0.83, 1.83) | 0.310 |
| GOT (U/l) | 2.20 (0.68, 7.11) | 0.190 |
| GPT (U/l) | 2.01 (0.64, 6.33) | 0.230 |
| INR | 4.75 (0.42, 54.38) | 0.210 |
| INR (adjusted for Coumarin intake) | 0.32 (0.01, 13.65) | 0.550 |
| CRP (mg/l) | 1.94 (0.99, 3.80) | 0.055 |
| NTproBNP (ng/l) | 2.23 (1.30, 3.85) | **0.003** |
| WBC (10^9/L) | 2.14 (0.27, 16.70) | 0.470 |

Logistic regression analysis results showing the associations of biomarkers with 30-day mortality. Parameters are all adjusted for the ES-II and firth corrected due to low event rates. The model based on INR is additionally adjusted for Coumarin intake. CI: Confidence interval, CRP: C-reactive protein, EuroSCORE: European System for Cardiac Operative Risk Evaluation, GOT: Glutamic Oxaloacetic Transaminase, GPT: Glutamate Pyruvate Transaminase, INR: International Normalized Ratio, NTproBNP: N-terminal pro B-type natriuretic peptide, WBC: white blood cells
